# Role of Circulating T Follicular Helper Cells and Stem-Like Memory CD4^+^ T Cells in the Pathogenesis of HIV-2 Infection and Disease Progression

**DOI:** 10.3389/fimmu.2021.666388

**Published:** 2021-04-16

**Authors:** Sivasankaran Munusamy Ponnan, K.K. Vidyavijayan, Kannan Thiruvengadam, Nancy Hilda J, Manikannan Mathayan, Kailapuri Gangatharan Murugavel, Luke Elizabeth Hanna

**Affiliations:** ^1^ Department of HIV/AIDS, National Institute for Research in Tuberculosis (Indian Council of Medical Research), Chennai, India; ^2^ Centre for Infectious Disease Research, Indian Institute of Science, Bangalore, India; ^3^ Centre for Drug Discovery and Development, Sathyabama Institute of Science and Technology, Chennai, India; ^4^ Division of Immunology, YRG Centre for AIDS Research and Education, Chennai, India

**Keywords:** HIV-2, ART, CTL, TSCM cells, TFH cells, CXCR5+ CD8+ cells, B cells

## Abstract

CD4^+^ T cells are critical players in the host adaptive immune response. Emerging evidence suggests that certain CD4^+^ T cell subsets contribute significantly to the production of neutralizing antibodies and help in the control of virus replication. Circulating T follicular helper cells (Tfh) constitute a key T cell subset that triggers the adaptive immune response and stimulates the production of neutralizing antibodies (NAbs). T cells having stem cell-like property, called stem-like memory T cells (Tscm), constitute another important subset of T cells that play a critical role in slowing the rate of disease progression through the differentiation and expansion of different types of memory cell subsets. However, the role of these immune cell subsets in T cell homeostasis, CD4^+^ T cell proliferation, and progression of disease, particularly in HIV-2 infection, has not yet been elucidated. The present study involved a detailed evaluation of the different CD4^+^ T cell subsets in HIV-2 infected persons with a view to understanding the role of these immune cell subsets in the better control of virus replication and delayed disease progression that is characteristic of HIV-2 infection. We observed elevated levels of CD4^+^ Tfh and CD4^+^ Tscm cells along with memory and effector T cell abundance in HIV-2 infected individuals. We also found increased frequencies of CXCR5^+^ CD8^+^ T cells and CD8^+^ Tscm cells, as well as memory B cells that are responsible for NAb development in HIV-2 infected persons. Interestingly, we found that the frequency of memory CD4^+^ T cells as well as memory B cells correlated significantly with neutralizing antibody titers in HIV-2 infected persons. These observations point to a more robust CD4^+^ T cell response that supports B cell differentiation, antibody production, and CD8^+^ T cell development in HIV-2 infected persons and contributes to better control of the virus and slower rate of disease progression in these individuals.

## Introduction

Acquired immunodeficiency in humans is caused by two types of human immunodeficiency virus (HIV), namely HIV-1 and HIV-2 (1). HIV-2 is less pathogenic than HIV-1, the course of disease is slower, and the proportion of disease controllers is significantly greater with HIV-2 infection (2). Unlike the case in HIV-1 infection, reduction in CD4^+^ T cells is much slower in HIV-2 infection (2). Several studies have documented the significance of antiviral CD4^+^ as well as CD8^+^ T cells in the control of infection in HIV-1 non-progressors and HIV-2 slow progressors (3–7). Studies have also shown differences in levels of expression of immune activation as well exhaustion markers including HLA-DR, PD1, CCR5, SAMHD1, Blimp-1 and TRIM5α on CD4^+^ T cell subsets in HIV-1 and HIV-2 infected individuals (8). Other factors that have been implicated in slower disease progression associated with HIV-2 infection include higher frequencies of virus-specific highly differentiated polyfunctional T cells and high titers of neutralizing antibodies (7, 9). However, the role of recently identified CD4+ T cell subsets like the T follicular helper cells and stem cell like memory T cells that are known to play a critical role in the control of HIV infection have not been investigated in the context of HIV-2 infection.

B cell-helping follicular T helper cells (Tfh cells) are reported to be an important subset of T cells involved in the production of high titers of neutralizing antibodies during the course of HIV disease as well as vaccination (10, 11). Earlier studies have documented the central role of Tfh cells in the control of HIV infection in long-term non-progressors (LTNPs) (8). A recent study found that Tfh cells constitute major viral reservoirs even in HIV-2 infected individuals with undetectable viremia and preserved blood CD4^+^ T cell counts (12). Since the last decade, T cells having stem cell-like properties (T_SCM_ cells) have gained much focus. The self-renewal capacity and longer survival period of T_SCM_ cells gives them the opportunity to differentiate into effector T cells (13). Increased frequency of T_SCM_ cells has also been reported in HIV-vaccinated individuals as well as elite controllers (EC) (14, 15). While there is evidence to suggest that T_SCM_ cells are essential for differentiation and enrichment of mature central effector (CE), effector memory (EM), and terminal effector (TE) cells in HIV-1 infected individuals (16–18), the possible role of these cells in HIV-2 infection has not been well explored.

It has been reported that the total number of B cells in peripheral blood also increases with an increase in CD4^+^ T cell count after initiation of ART (19, 20), since the CD4^+^ T cells connect the cellular and humoral arms of the immune system. Studies have reported that memory B cell subsets are significantly expanded in HIV-2 infected persons as compared to HIV-1 infected individuals irrespective of their treatment status (21–23). Our data also demonstrate a significant expansion in the antigen-specific CD4^+^ T cell subset in HIV-2 infected individuals, and suggest that these cells could support the activation of plasmablasts, atypical and memory B cells, as well as polyfunctional T cell and antigen-presenting cell (APC) activity (24), thus providing the basis for a robust host immune response against the pathogen. Similarly, studies on NK cells in HIV infection have critically acclaimed their role in antiviral activity (25–27). Functional and genetic studies have demonstrated the role of NK cells in slowing down disease progression (28). However, as infection progresses, NK cell subsets undergo phenotypic and functional abnormalities resulting in failure to control viral movement (28). While a large number of studies have focused on understanding the phenotype and function of NK cells in HIV-1 infection, very little is known about the role of these cell subsets in HIV-2 disease and their contribution to viral control.

The current study was undertaken to understand the role of Tfh cells, T_SCM_ cells, follicular homing CD8^+^ T cells, as well as B cells and NK cells in determining the course of disease progression in HIV infection. We found elevated levels of CD4^+^ as well as CD8^+^ T cells that are phenotypically unique and express higher levels of markers pertaining to T stem cell--likeness and follicular homing phenotype, as well as CXCR5 on their surface in HIV-2 infected persons. Furthermore, we observed a higher frequency of memory B cells and NK cells in these individuals. Our observations throw light on some of the critical CD4^+^ and CD8^+^ T cell subsets and immune responses associated with better protection against disease progression in HIV-infected individuals and suggest that a successful HIV vaccine should elicit some of these key immune responses in order to confer protection in vaccine recipients.

## Materials and Methods

### Ethics Statement

The study protocol was approved by the Scientific Advisory Committee of the ICMR-National Institute for Research in Tuberculosis (NIRT), Chennai, India, and the study was conducted in accordance with Good Clinical Laboratory Practice (GCLP) guidelines. The study protocol was also reviewed and approved by the Institutional Ethics Committee of ICMR-NIRT (TRC IEC No: 2009009) and the Institutional Review Board of the Y. R. Gaitonde Centre for AIDS Research and Education (YRG IRB No: 279), Chennai, India.

### Study Participants

The study was carried out on samples collected during the period 2016-2018. Sample collection was performed at YRG CARE, one of the largest tertiary referral HIV Care Centres in southern India, providing medical care and support for more than 22,000 patients, and the study was conducted at ICMR-NIRT. The study population comprised of 4 groups of individuals: (i) HIV-2 infected persons (HIV-2; n=37), (ii) HIV-1 infected individuals on antiretroviral therapy (HIV-1+ART+; n=10),(iii) HIV-infected individuals naïve to ART (HIV-1+ART-; n=10), and (iv) HIV--unexposed uninfected healthy women (HU; n=35). Individuals in groups 1 & 2 received ZDV (Zidovudine) + 3TC (Lamivudine) + NVP (Nevirapine) or TDF (Tenofovir) + 3TC (Lamivudine) + EFV (Efavirenz) as per the National Technical Guidelines on Antiretroviral Treatment (NACO, 2018). In addition, we also included a group of HIV exposed seronegative (HESN) individuals who were HIV-uninfected female spouses of HIV-1 seropositive men (HIV discordant couples; n=35) from one of our earlier studies (unpublished data) for comparison. Enrolment into the study required the willingness of participants to provide written informed consent for specimen collection and storage. All HIV-2 infected persons were free of HIV-1 co-infection at the time of sampling (**Table 1**). Diagnosis and confirmation of HIV-2 infection was based on serological and molecular diagnostic tests. Serological testing was done using the Retro quick rapid test (Qualpro Diagnostics) and HIV-1/2 Tridot (J. Mitra). Samples were further tested using molecular tests to confirm the absence of HIV-1 co-infection with negative detection in HIV-1 DNA-PCR and positive detection in HIV-2 gene-specific PCR.

**Table 1:** Demographic characteristics of Study Populations Groups.

	HIV-2	HU	HIV+ART+	HIV+ART-	HE SN
	N=37	N=35	N=10	N=10	N=35
Age mean (range)	43 (18-57)	33(22-42)	34(32-39)	35(30-48)	36(27-42)
STDs (BV, CT, NG)	0	0	0	0	0
ART details	37(100%)	0	10(100%)	NA	NA
Viral Load, Log10 copies/mL, mean (SD)	NA	NA	2.14	4.4943(0.9036)	NA
CD4 Count at treatment initiation (cells/mL), median (IQR)	617(33-3097)	NA	392(289-492)	NA	NA

BV, Bacterial Vaginitis; CT, Chlamydia trachomatis; NG, Neisseria gonorrhoeae; HESN, HIV exposed seronegative; HIV+ART+, HIV-infected women on ART; HIV+ART-, HIV-infected women naïve to ART; HU, HIV unexposed seronegative controls.

### Isolation and Cryopreservation of PBMC

Ten milliliters of blood was collected from all study participants by venepuncture in a green top VACUTAINER® containing sodium heparin as the anticoagulant and used for the isolation of peripheral blood mononuclear cells (PBMC). PBMC were isolated by density gradient centrifugation and cryopreserved at <-190°C.

### Phenotypic Analysis of Immune Cell Subsets

Cryopreserved PBMC obtained from the study participants were used for immunophenotyping by flow cytometry. Briefly, two million cells were washed with FACS buffer and stained with Live/Dead Fixable Aqua blue dye (Invitrogen). The following cocktail of monoclonal antibodies were used to enumerate the different cell types: T follicular helper and Treg panel: CD3-APC H7 (SK7), CD4-BUV 737 (SK3), CD8-APC R 700 (RPA-T8), CD45RO BUV395 (UCHL-1),CCR7-PEcy7 (G043H7), CXCR3-PERCP Cy5.5 (IC6), CXCR5-BB515 (RF8B2), PD-1-PE (EH12.1), CD25-APC (A251) and CD127-PECF594 (HIL.7R.M21); B cell panel: CD3-APC H7(SK7), CD38-APC (HIT2), CD20-PE (HIB19), CD19-BUV395 (3H7), IgD-BUV737 (IA6.2), CD27-BB515 (0323; Memory T cell and T_SCM_ panel: CD3-APCH7 (SK7), CD8-APCR700 (RPA.T8), CD4-BUV737 (RPA-T4), CD45RO-BUV395 (UCHL-1), CCR7-PEcy7 (G043H7),CD95-PECF594 (DX2), CD28-APC (28.2), CD122-PE (MIKB2). NK cell panel: CD3-APCH7 (SK7), CD56-APC(B519), CD16-BUV737 (3CG8), CD95- PECF594 (DX2), NKG2D-PERCP CY5.5 (1D11). Cells were stained with the antibodies for 20 minutes at 4°C (antibody and clone description are provided in **Table S1**). About 2 × 10^6^ cells were stained for each panel. After staining, the cells were washed, fixed with BD Cytofix (2% paraformaldehyde), and analyzed on a FACS ARIA III SORP flow cytometer (Becton Dickinson). A minimum of 1,000,000 total events were acquired, and data were analyzed using FlowJo software, version 10.5 (Tree Star Inc., Ashland, Oregon, USA).

### Statistical Analysis

Statistical analyses were performed using GraphPad Prism, version 7.05 (GraphPad Software, Inc., CA). Values are presented as median and interquartile range. Percentage frequency of the immune cell subsets like memory T cells, follicular helper-like CD8^+^ T cells (CXCR5+ CD8^+^ T cells), T_SCM_ cells, B cells and NK cells were compared between the HIV-2, HIV-1, HESN and HU groups using Kruskal-Wallis test, followed by subgroup analysis using Dunn’s multiple comparison test. Correlation analysis was performed to determine the relationship between the frequency of different immune cell types and broadly neutralizing antibodies titer. For all analyses, differences were considered significant if the p-value was <0.05.

## Results

### Expansion of Circulating T Follicular Helper Cells, Stem-Like Memory CD4^+^ T Cells, and CXCR5^+^CD8^+^ T Cells in HIV-2 Infected Individuals

**Figure 1:**
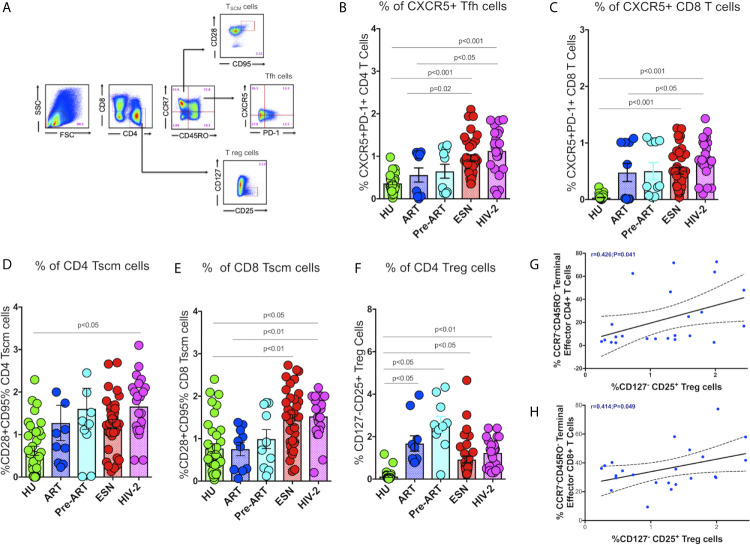
Distribution of Tfh cells and stem-like memory T cells in the study population. **(A)** Representative flow plots for enumeration of Tfh cells, CXCR5^+^ T cells, Tscm cells and regulatory T cells. **(B)** Frequency of CXCR5^+^ CD4^+^ Tfh cells in the HU, HIV-1+ART+, HIV-1+ART-, HESN and HIV-2 groups. **(C)** Frequency of CXCR5^+^ CD8^+^ T cells in the HU, HIV-1+ART+, HIV-1+ART-, HESN and HIV-2 groups. **(D)** Frequency of CCR7-CD28+CD95+ stem-like memory CD4^+^ T cells in the HU, HIV-1+ART+, HIV-1+ART-, HESN and HIV-2 groups. **(E)** Cumulative frequency of CCR7-CD28+CD95+ stem-like memory CD8^+^ T cells in the HU, HIV-1+ART+, HIV-1+ART-, HESN and HIV-2 groups. **(F)** Frequency of CD127- CD25+ regulatory T cells in the HU, HIV-1+ART+, HIV-1+ART-, HESN and HIV-2 groups. Scatter dot plots summarize the % frequency of total CXCR5+ T cells, Tscm cells and Treg cells (median, 1st and 3rd quartiles). P values were calculated using the K-Wallis test. Sub-group analysis was performed using Dunn’s test. **(G)** Correlation between frequency of CCR7-CD45RO- Terminal Effector Memory CD4^+^T cells and frequency of CD25+127- CD4^+^ T Regulatory cells in HIV-2 infected individuals. **(H)** Correlation between frequency of CCR7-CD45RO- Terminal Effector Memory CD8^+^ T cells and frequency of CD25+127- CD4^+^ T regulatory cells in HIV-2 infected individuals. (Note: HU: HIV unexposed seronegative controls, n = 35; HIV-1+ART+: HIV-1-infected women on ART, n = 10; HIV-1+ART-: HIV-infected women naïve to ART, n = 10; HESN: HIV exposed seronegative individuals, n = 35; HIV-2: HIV-2 infected persons, n = 37).

Recent studies suggest that follicular CXCR5**^+^** CD8^+^ T cells and CD8^+^ stem-like memory cells are involved in the control of HIV/SIV infection (29, 30). We therefore, evaluated the frequencies of these T cell subsets in HIV-2 infected individuals and compared it with that seen in HIV-exposed seronegative (HESN) individuals, HIV-1 infected persons on antiretroviral treatment (HIV-1+ART+) as well as those not on treatment (HIV-1+ART-), and HIV-unexposed healthy individuals (HU). Polychromatic flow cytometry was used to enumerate the different T cell subsets, including circulating Tfh cells, CXCR5 expressing CD8^+^ T cells, stem-like memory T cells, and regulatory T cells. Tfh and CXCR5 expressing CD8^+^ T cells were defined using CXCR5 and PD-1, T stem-like memory cells were identified using CD28 and CD95 expression on naïve T cells, and T regs were identified based on the expression of CD25 on CD127^-^ CD4^+^ T cells (**Figure 1A**).

We found significantly elevated numbers of CD4^+^ Tfh cells in HIV-2 infected individuals (median: 1.21%; range: 0.70-1.56%) as compared to HIV-1 infected persons (median: 0.58%; range: 0.04-1.04%; p<0.05) as well as unexposed healthy individuals (median: 0.37%; range: 0.23-0.44%; p<0.001) (**Figure 1B**). Incidentally, we also found higher numbers of CD4^+^ Tfh cells in the ESN group (median: 0.86%; range: 0.61-1.27%) as compared to the HIV-infected (median: 0.58%; range: 0.04-1.04%; p=0.02) and HIV-unexposed healthy control groups (median: 0.37%; range: 0.23-0.44%; p<0.001). Further, we found significantly higher frequencies of CXCR5^+^CD8^+^ T cells in HIV-2 infected individuals (median: 0.90%; range: 0.61-1.10%) as compared to HIV-1 infected individuals on ART (median: 0.32%; range: 0.01-1.01%; p<0.05) as well as HIV-unexposed healthy controls (median: 0.01; range: 0.01-0.04%) (p<0.001) (**Figure 1C**). Collectively, our findings reveal significant expansion of critical T cell subsets that help boost the host immune response leading to better control of the virus and slower course of disease progression in HIV-2 infected individuals.

Earlier studies have reported that the self-renewing and multipotent Tscm cells mature into memory cells or reservoirs of effector T lymphocytes, which continue to persist in the host even in the absence of antigen (31). Hence, expansion of Tscm cells appears to be a very critical component in the control of HIV infection. Very interestingly, we found significantly increased numbers of Tscm cells in both the CD4^+^ and CD8^+^ T cell compartments in HIV-2 infected individuals as compared to HIV-1-infected persons (**Figures 1D, E**). The median frequency of circulating CD4^+^ Tscm cells in the HIV-2 group was 1.80% (range: 1.10-2.10%), HIV-1 infected group on ART was 0.8% (range: 0.31-1.80%), HIV infected group naïve to ART was 1.20% (range: 0.70-2.10%), and unexposed healthy groups was 0.63% (range: 0.01-1.45%) respectively. The median frequency of circulating CD8^+^ Tscm cells in the HIV-2 group was 1.70% (range: 1.21-2.21%), HIV-1 infected group on ART was 0.877% (range: 0.26-1.23%), HIV infected group naïve to ART was 0.82% (range: 0.21-1.81%), and unexposed healthy groups was 0.56% (range: 0.23-1.17%). Another striking observation was the presence of a significantly higher frequency of CD8^+^ Tscm cells in the HESN population (median 1.26%; range: 0.71-1.93%) as compared to the HIV-1 infected and HU groups.

In contrast, we observed a decreased frequency of Treg cells in the HIV-2 and HESN groups as compared to the other groups. The ART-naive HIV-infected group had a significantly higher proportion of CD4^+^ Treg cells as compared to the other study groups (p<0.01) (**Figure 1F**). We further analyzed the correlation between the Tregs and memory T cell subsets and found an inverse correlation between Tregs and effector memory T cells in both CD4^+^ (r-0.4, p=0.04) and CD8^+^ (r-0.4, p=0.05) T cell compartments in HIV-2 infected individuals (**Figures 1G, H**). These findings clearly indicate that increased frequency of Tregs help maintain immune homeostasis by controlling the exaggerated immune response and promoting the development of long-lived memory cells during HIV-2 infection.

### Increased Frequency of Memory B Cells and Plasmablast B Cells in HIV-2 Infection

**Figure 2:**
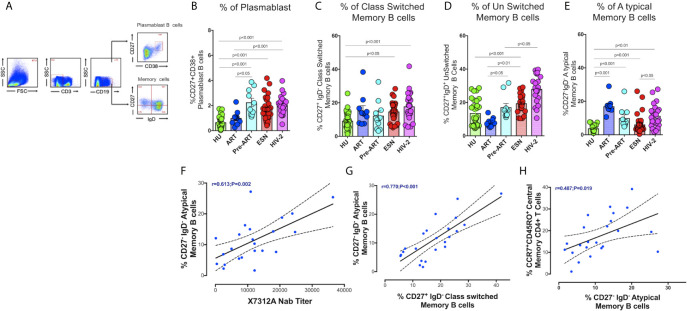
Distribution of Memory B cells and Plasmablast B cells in the study groups. **(A)** Representative flow plots for enumeration of memory B cells and Plasmablast B cells (CD27+CD38+ expression on B cells) among the HU, HIV-1+ART+, HIV-1+ART-, HESN and HIV-2 groups. **(B)** Frequency of CD27+CD38+ Plasmablast B cells in the HU, HIV-1+ART+, HIV-1+ART-, HESN and HIV-2 groups. **(C)** Frequency of CD27+IgD- Class Switched Memory B cells in the HU, HIV-1+ART+, HIV-1+ART-, HESN and HIV-2 groups. **(D)** Frequency of CD27-IgD- unswitched Memory B cells in HU, HIV-1+ART+, HIV-1+ART-, HESN and HIV-2 groups. **(E)** Frequency of CD27+IgD+ Atypical Memory B cells in HU, HIV-1+ART+, HIV-1+ART-, HESN and HIV-2 groups. The scatter dot plots summarize the % frequency of total memory B cells and Plasmablast cells (median, 1st and 3rd quartiles). P values were calculated using the K-Wallis test. Sub-group analysis was performed using Dunn’s test. **(F)** Correlation between frequency of CD27-IgD- Atypical Memory B cells and X7312A neutralizing antibody titers in HIV-2 infected individuals. **(G)** Correlation between frequency of CD27-IgD- Atypical memory B cells and frequency of CD27+IgD- Class Switched Memory B cells in HIV-2 infected individuals. **(H)** Correlation between frequency of CCR7+ CD45RO+ Central Memory CD4^+^ T cells and frequency of CD27-IgD- Atypical Memory B cells in HIV-2 infected individuals. (HU, HIV unexposed seronegative controls, HIV-1+ART+: HIV-1-infected women on ART; HIV-1+ART-: HIV-1-infected women naïve to ART; HESN: HIV exposed seronegative individuals; HIV-2: HIV-2 infected persons).

We analyzed the frequency of different memory B cell subsets in the study groups. Surface markers CD27 and CD38 were used to identify plasmablast B cells (CD19+CD38+CD27+). Memory B cell subsets were distinguished based on IgD and CD27 expression as unswitched memory B cells (IgD+CD27+), class switched memory B cells (IgD- CD27+), atypical memory B cells (IgD-CD27-), and naive B cells (IgD+CD27-) as described by Kaminski et al. (32) (**Figure 2A**). We found a higher frequency of short-lived antibody-secreting plasmablast cells in the HIV-2 positive (median:1.91%; range 1.50 - 2.40%) as well as HESN (median:1.44%; range: 1.05 - 2.14%) groups as compared to treated HIV-1 infected and healthy uninfected groups (median: 1.01%; range: 0.64 -1.69%; p<0.001). Numbers of plasmablast cells were also significantly higher in ART-naïve HIV-1-infected individuals as compared to treated individuals (median:1.95%; range: 1.30- 3.61% p<0.05) (**Figure 2B**). These observations suggest that HIV infection, in general, is associated with an increase in the numbers of antibody-secreting plasmablast cells that further differentiate into long-lived plasma cells and contribute to better control of infection, but their role in slowing down disease progression remains to be clarified.

The HIV-2 and HESN groups were found to possess significantly higher frequencies of class-switched memory B cells and IgM memory B cells (unswitched) as compared to the HIV-1 and HU groups (p<0.001). The median frequency of class-switched memory B cells was 18.2% (range: 12.5 - 23.2%) and unswitched memory B cells was 27.6% (range: 22.8 - 35.4%) in the HIV-2 group (**Figures 2C, D**). We observed a significantly higher proportion of atypical memory B cells in both HIV-1+ART+ and HIV-2 groups as compared to the other groups (**Figure 2E**).

We found a direct correlation between the frequency of atypical memory B cells and the titer of neutralizing antibodies in HIV-2 infected persons (r=0.61, p=0.002) (**Figure 2F**) (neutralizing antibody titres of HIV-2 infected persons is provided in Supplementary Table 1). Levels of atypical memory B cells also correlated positively with the frequency of class-switched memory B cells and central memory CD4^+^ T cells (r=0.48, p<0.019 and r=0.77, p<0.001 respectively) (**Figures 2G, H**). These observations provide further evidence to suggest that early HIV-2 infection is associated with increased CD4^+^ T cell help for B cell differentiation resulting in the development of increased numbers of memory B cell subsets as well as antibody-secreting plasma B cells, thus constituting a robust immune response to fight the infection.

### Increased Frequency of Effector Memory CD4^+^ and CD8^+^ T Cells in HIV-2 Infected Individuals

We examined the distribution of memory CD4^+^ and CD8^+^ T cells in HIV-2 infected individuals, given the potential role of these cell types in slowing down disease progression. Effector memory cells are characterized by the expression of C-C chemokine receptor type-7 and CD45RO on their cell surface. Based on their function, 4 distinct populations of effector cells have been described by Gattinoni et al. (31). These include the central memory (CM) T cells defined as CD3+CD4+/CD8+CD45RO+CCR7+ cells, effector memory (EM) T cells defined as CD3+CD4+/CD8+CD45RO+CCR7- cells, terminal effector (TE) T cells defined as CD3+CD4+/CD8+CD45RO-CCR7- cells and naïve T cells (TN) defined as CD3+CD4+/CD8+CD45RO-CCR7+ cells.

**Figure 3:**
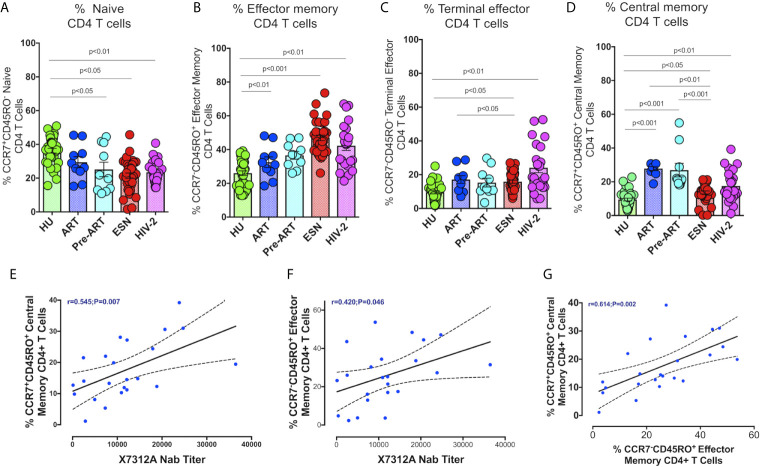
Distribution of CD4^+^ Memory T cell subsets in the study groups. Graphical representation showing the enumeration of CD4^+^ Memory T cells subsets defined using CCR7 and CD45RO expression. **(A)** Frequency of CCR7+CD45RO- naïve CD4^+^ T cells in the HU, HIV-1+ART+, HIV-1+ART-, HIV-2 and HESN groups. **(B)** Frequency of CCR7-CD45RO+ Effector Memory CD4^+^ T cells in the HU, HIV-1+ART+, HIV-1+ART-, HIV-2 and HESN groups. **(C)** Frequency of CCR7-CD45RO- Terminal Effector Memory CD4^+^ T cells HU, HIV-1+ART+, HIV-1+ART-, HIV-2 and HESN groups. **(D)** Frequency of CCR7+CD45RO+ Central Memory CD4^+^ T cells in the HU, HIV-1+ART+, HIV-1+ART-, HIV-2 and HESN groups. The scatter dot plots summarize the % frequency of total memory T cells (median, 1st, and 3rd quartiles). P values were calculated using the K-Wallis test. Sub-group analysis was performed using Dunn’s test. **(E)** Correlation between frequency of CCR7+ CD45RO+ Central memory CD4^+^ T cells and X7312A neutralizing antibody titers in HIV-2 infected individuals. **(F)** Correlation between frequency of CCR7-CD45RO+ Effector Memory CD4^+^ T cells and X7312A neutralizing antibody titers in HIV-2 infected individuals. **(G)** Correlation between frequency of CCR7+ CD45RO+ Central Memory CD4^+^ T cells and frequency of CCR7-CD45RO+ Effector Memory CD4^+^ T cells in HIV-2 infected individuals. (Note: HU: HIV unexposed seronegative controls, HIV-1+ART+: HIV-1-infected women on ART; HIV-1+ART-: HIV-1-infected women naïve to ART; HESN: HIV exposed seronegative individuals; HIV-2: HIV-2 infected persons).

The proportion of CD4^+^ and CD8^+^ naïve T cells was significantly lower in the HIV-2, HESN, HIV-1+ART+ and HIV-1+ART- groups as compared to the HU group (**Figure 3A**). On the other hand, the frequency of CD4^+^ effector memory T cells and terminal effector T cells was significantly higher in the HIV-2 and HESN groups (**Figures 3B, C**). While the median frequency of CD4^+^ terminal effector T cells was 18.6 % (range: 12.7- 36.5%) in the HIV-2 group and 14.31% (range: 11.40 - 20.21%) in the HESN group, the median frequency of CD4^+^ effector memory T cells was 43.6% (range: 31.5 - 52.3%) in the HIV-2 group and 44.1% (range: 39.5 - 55.1%) in the HESN group. Earlier studies have also reported a progressive expansion of terminal effector T cells during viral infections, including HIV-1 infection (33–35). We also found that the proportion of cytotoxic function exhibiting terminal effector T cells was very high in HIV-2 infected individuals as compared to HIV-1 infected persons and healthy controls. On the other hand, the proportion of CD4^+^ central memory T cells was higher in the HIV-1 group as compared to the HIV-2 group (**Figure 3D**).

We examined the correlation between the frequency of memory T cell subsets and neutralizing antibody titers in HIV-2 infected individuals. Interestingly, we found a positive correlation between the frequency of CD4^+^ central memory cells as well as effector memory T cells with X731A neutralizing antibodies titer (r=0.54, p=0.007, and r=0.42, p=0.046 respectively) in HIV-2 infected individuals (**Figures 3E, F**). In addition, we noticed a correlation between CD4^+^ central memory T cells and CD4^+^ effector memory T cells (r-0.61, p=0.002) (**Figure 3G**) in the study population.

**Figure 4:**
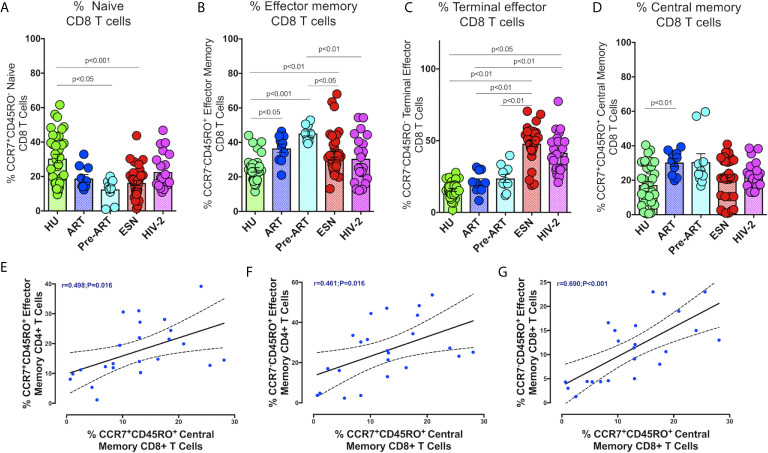
Distribution of CD8^+^memory T cells responses in the study groups. Graphical representation of the enumeration of CD8^+^memory T cells subsets defined using CCR7 and CD45RO expression. **(A)** Frequency of CCR7+CD45RO- naïve CD8^+^ T cells in the HU, HIV-1+ART+, HIV-1+ART-, HIV-2 and HESN groups. **(B)** Frequency of CCR7-CD45RO+ Effector Memory CD8^+^ T cells in the HU, HIV-1+ART+, HIV-1+ART-, HIV-2 and HESN groups. **(C)** Frequency of CCR7-CD45RO- Terminal Effector Memory CD8^+^ T cells HU, HIV-1+ART+, HIV-1+ART-, HIV-2 and HESN groups. **(D)** Frequency of CCR7+CD45RO+ Central Memory CD8^+^ T cells in the HU, HIV-1+ART+, HIV-1+ART-, HIV-2 and HESN groups. The scatter dot plots summarize the % frequency of total memory T cells (median, 1st, and 3rd quartiles). P values were calculated using the K-Wallis test. Sub-group analysis was performed using Dunn’s test. **(E)** Correlation between frequency of CCR7-CD45RO+ Effector Memory CD4^+^ T cells and frequency of CCR7+CD45RO+ Central Memory CD8^+^ T cells in HIV-2 infected individuals. **(F)** Correlation between frequency of CCR7-CD45RO+ Effector Memory CD4^+^ T cells and frequency of CCR7-CD45RO+ Effector Memory CD8^+^ T cells in HIV-2 infected individuals. **(G)** Correlation between frequency of CCR7-CD45RO+ Effector Memory CD4^+^ T cells and frequency of CCR7+CD45RO+ Central Memory CD8^+^ T cells in HIV-2 infected individuals. (Note: HU: HIV unexposed seronegative controls, HIV-1+ART+: HIV-1-infected women on ART; HIV-1+ART-: HIV-1-infected women naïve to ART; HESN: HIV exposed seronegative individuals; HIV-2: HIV-2 infected persons).

We also examined the distribution of CD8^+^ memory T cells in HIV-2 infected individuals. As seen with CD4^+^ T cells, naïve CD8^+^ T cells were present at significantly lower levels in the HIV-1 and HIV-2 infected groups as compared to the healthy controls (**Figure 4A**). On the other hand, CD8^+^ terminal effector memory T cells were significantly higher in the HIV-2 and HESN groups as compared to the other groups (p<0.05). The median frequency of CD8^+^ terminal effector T cells was 40.3% (range: 30.4 - 49.4%) in the HIV-2 group and 48.30% (range: 44.31-55.30%) in the HESN group (**Figure 4C**). In contrast, HIV-1-infected persons had a significantly higher proportion of CD8^+^ effector and central memory T cells (p<0.001) (**Figure 4B**). The median frequency of CD8+ effector memory T cells was 44.6% (range: 40.6 - 49.9%) in the HIV-1+ART- group and 38.6% (range: 29.5 - 43.3%) in the HIV-1+ART+ group. The median frequency of CD8^+^ central memory T cells was 24.4% (range: 20.7 - 34.8%) in the HIV-1+ART- group and 32.2% (range: 22.4 - 36.1%) in the HIV-1+ART+ group (**Figure 4D**).

In HIV-2 infected individuals, correlation analysis revealed a significant correlation between the frequency of CD4^+^ effector memory T cells and CD8^+^ central memory T cells (r=0.49, p=0.016) (**Figure 4E**). Similarly, CD4^+^ effector memory T cells correlated significantly with levels of CD8^+^ effector memory T cells and CD8^+^ central memory T cells (r=0.46, p=0.016 and r=0.48, p=0.020) (**Figure 4F**). Levels of CD8^+^ central memory T cells correlated positively with CD8^+^ effector memory T cells (r-0.69, p<0.001) (**Figure 4G**).

### Increased Frequency of NKG2D Expressing CD56 Negative CD16+ NK Cells in HIV-2 Infected Persons

**Figure 5:**
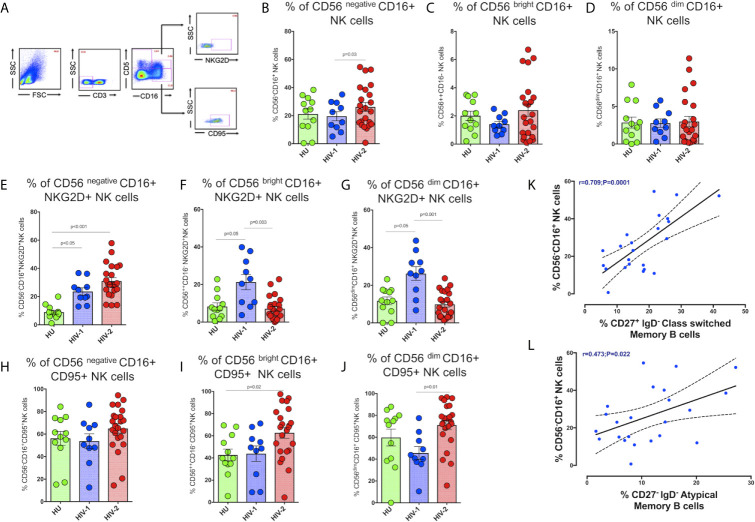
Distribution of NK cell subsets in the study population. **(A)** Representative flow plots showing the enumeration of NK cell subsets based on CD16 and CD56 expression in the study population (HU N=35, HIV-1+ART+ N=10, and HIV-2 N=37). **(B)** Frequency of CD56 negative CD16+ NK cells in the HU, HIV-1+ART+ and HIV-2 groups. **(C)** Frequency of CD56 bright CD16+ NK cells in the HU, HIV-1+ART+ and HIV-2 groups. **(D)** Frequency of CD56 dim CD16+ NK cells in the HU, HIV-1+ART+ and HIV-2 groups. **(E)** Frequency of CD56 negative CD16+NKG2D+ NK cells in the HU, HIV-1+ART+ and HIV-2 groups. **(F)** Frequency of CD56 bright CD16+NKG2D+ NK cells in the HU, HIV-1+ART+ and HIV-2 groups. **(G)** Frequency of CD56 dim CD16+NKG2D+ NK cells in the HU, HIV-1+ART+ and HIV-2 groups. **(H)** Frequency of CD56 negative CD16+CD95+ NK cells in the HU, HIV-1+ART+ and HIV-2 groups. **(I)** Frequency of CD56 bright CD16+CD95+ NK cells in the HU, HIV-1+ART+ and HIV-2 groups. **(J)** Frequency of CD56 dim CD16+95+ NK cells in the HU, HIV-1+ART+ and HIV-2 groups. The scatter dot plots summarize the % frequency of NK cell subsets with differential expression of CD56 and CD16 (median, 1st and 3rd quartiles). P values were calculated using the K-Wallis test. Sub-group analysis was performed using Dunn’s test. **(K)** Correlation between the frequency of CD56 negative CD16+ NK cells and frequency of CD27+IgD- Class Switched Memory B cells in HIV-2 infected individuals. **(L)** Correlation between the frequency of CD56 negative CD16+ NK cells and frequency of CD27-IgD- Atypical Memory B cells in HIV-2 infected individuals. (Note: HU: HIV-unexposed seronegative controls; HIV-1+ART+: HIV-1-infected women on ART; HIV-2: HIV-2 infected individuals.

NK cell-mediated ADCC is thought to be an essential mechanism involved in controlling HIV infection. Several studies have described NK cell activation and cytolytic function in HIV-1 infected individuals as well as in long-term non-progressors (LTNP) and elite controllers (EC) (36–39). However, the distribution of NK cells and their profile in HIV-2 disease remains mostly undefined. To address this knowledge gap, we characterized the population of NK cell subsets expressing the activation receptor NKG2D and the exhaustion marker CD95 receptor in the study population (**Figure 5A**).

We observed significantly higher frequencies of CD56^negative^ CD16^+^ NK cells in HIV-2 infected individuals as compared to HIV-1-infected persons and unexposed healthy individuals (p=0.036) (**Figure 5B**). The median frequency of CD56^negative^ CD16^+^ NK cells in the HIV-2 group was 25% (range:14.1 - 38.5%) and 12.9% (range: 10.4 - 17.1%) in the HIV-1 group. On the other hand, there was no difference in the proportion of CD56^dim^ CD16^+^ NK cells as well as CD56^bright^ CD16^+^ NK cells between the groups (**Figures 5C, D**). However, earlier studies have documented a significant reduction in the proportion of mature CD56^dim^ NK cells as well as immature CD56^bright^ NK cells in HIV-1 infection (40), with a concomitant increase in the numbers of CD56^negative^ NK cells (41). Our study demonstrates a greater increase in the proportion of CD56^negative^ NK cells than CD56^bright^ and CD56^dim^ NK cells in HIV-2 infected individuals.

We also found significantly higher numbers of CD56^negative^ NK cells expressing the NKG2D activating receptor in HIV-2 infected individuals than in HIV-1 and HU individuals (p<0.001 and p<0.05, respectively) (**Figure 5E**). On the other hand, HIV-1 infected individuals had significantly higher numbers of NKG2D expressing CD56^bright^ and CD56^dim^ NK cells (**Figures 5F, G**). Similarly, CD95 expressing CD56^bright^ and CD56^dim^ NK cells were also significantly more in HIV-2 infected persons as compared to HIV-1-infected individuals and healthy unexposed controls. However, there was no difference between the groups with respect to CD95 expression on CD56^negative^ NK cells (**Figures 5H-J**).

We also examined the correlation between levels of CD56^negative^ NK cells and neutralizing antibodies titer as well as other memory T cells and B cell subsets in HIV-2 infected individuals. Interestingly, we found that levels of CD56^negative^ NK cells correlated significantly with levels of CD27^+^ IgD^-^ class-switched memory B cells as well as CD27^-^ IgD^-^ atypical memory B cells (r-0.70, p=0.0001 and r-0.47, p=0.022) (**Figures 5K, L**). Overall, our results indicate that the unconventional cytotoxic CD56^negative^ NK cells in HIV-2 infected individuals possess increased activation and reduced exhaustion properties and suggest a possible role for these cells in the slower course of disease progression that is characteristic of HIV-2 infection.

## Discussion

CD4^+^ T cells are the central mediators of the adaptive immune response. CD4^+^ T cell depletion is the hallmark of the pathogenesis of HIV-1 and HIV-2 infection (42). However, the host immune system continuously exchanges dying CD4^+^ T cells with naïve CD4^+^ T cells during the early stages of HIV infection (43). Compared to HIV-1, HIV-2 is less pathogenic and is associated with a long asymptomatic phase after infection, reduced viral load and slower decline of CD4^+^ T cells (44, 45). It may be assumed that elevated effector and memory cell differentiation could contribute to better control of viremia and slower disease progression in controllers of HIV infection (46). Earlier studies have reported a higher proportion of HIV-1 specific T_SCM_ cells and effector memory cells in elite controllers, suggesting a role for these cells in the resistance to HIV infection/disease progression (47, 48). Vigano et al. observed an inverse correlation between the frequency of total t_SCM_ cells and levels of plasma viremia in untreated HIV-1 infected individuals (49). A more recent study showed that, relative to effector CD4^+^ T cells, Tfh cells serve as the major viral reservoirs in HIV-2 infected persons (12). This makes it important to understand the role of different subsets of CD4^+^ T cells in HIV-2 infection in order to identify the correlates of delayed disease progression and slower course of disease associated with HIV-2 infection.

In the current study, we analyzed the role of key CD4^+^ T cell subsets in the control of HIV infection and disease progression in HIV-2 infected individuals by analyzing the expression of phenotypic and functional makers pertaining to memory differentiation, stem cell-likeness and follicular homing. We also investigated the correlation between neutralizing antibody titer and CD8^+^ memory T cell subsets in HIV-2 infected persons. We found significant expansion and enrichment of effector memory and terminal effector cells in the HIV-2 group. These findings are in line with those found in literature, where an increase in effector and terminal effector cell frequency has been reported in multiple viral infections, including HIV-1 infection (34, 50). Very interestingly, we found a significant association between central and effector memory CD4^+^ T cells and neutralizing antibody titer (neutralizing antibody data has been reported in an earlier publication from our group) (9). Just as in the case of CD4^+^ T cells, the frequency of effector memory and Terminal effector CD8^+^ T cells were also substantially higher in the HIV-2 infected group. These findings suggest that HIV-2 infection elicits a robust immune response capable of activating CD4^+^ T cells to provide continuous help to CD8^+^ T cells as well as B cells, leading to better control of viral replication and slower progression of disease.

Tscm cells are a small subset of T cells possessing self-renewal capabilities, that when stimulated via the T cell receptor, may divide into mature memory or effector T cells (49, 51). Many studies have consistently reported a close and robust association between the proliferation of effector cells and memory T cells and virus control in HIV infection (1, 2, 52). However, there is a lack of clarity in this line with regard to HIV-2 infection due to the limited number of studies in HIV-2 individuals. Interestingly, in the present study we found that HIV-2 infected persons had significantly higher levels of stem cell-like memory cells in both the CD4^+^ and CD8^+^ T cell compartments. Similarly, there was a significantly enriched population of Tfh cells in HIV-2-infected individuals as compared to HIV-1 infected persons. However, we found no correlation between levels of Tfh cells and B cells/neutralizing antibody titer. This could possibly due to the small sample size in the study. HIV-2 infected individuals also had significantly elevated numbers of circulating follicular homing CXCR5^+^CD8^+^ T cells as compared to HIV-1 infected individuals.

HIV-1 natural controllers are known to possess high levels of CXCR5^+^ CD8^+^ Tscm cells, which correlate inversely with the viral load (14). We found higher frequencies of Tscm and CXCR5^+^ CD8^+^ T cells with effector memory phenotype in HIV-2 individuals as compared to HIV-1 infected persons. All this evidence suggests a plausible role for HIV-2-specific Tscm cells and Tfh cells in slowing down disease progression in HIV-2 infected persons. To the best of our knowledge, the present study constitutes the first research to record elevated frequencies of Tfh cells, Tscm cells, and follicular homing CXCR5^+^ CD8^+^ T cells in an HIV-2 cohort from India. However, the mechanisms by which these cells reduce the viral load in HIV-2 individuals requires further investigation.

In healthy individuals, most B cells in peripheral blood are either resting naive B cells or classical type memory B cells that express either switched or unswitched antibody isotypes (IgG, IgE, and IgA, or IgM and IgD respectively) (32). In chronic HIV infection, absolute numbers of both classical and memory B cells are decreased in the peripheral circulation (53). Moir et al. reported that ART-naïve HIV-1 individuals with chronic infection had a highly expanded population of immature/transitional B cells, whereas in early infection, plasmablasts and atypical memory B cells were more prevalent (19). A previous study showed that plasmablasts and class-switched memory B cells were induced upon vaccination in a phase-1 MVA and ADVAX prime-boost vaccine trial carried out in India (54). As with other studies, we too documented an expansion of peripheral plasmablasts as well as atypical exhausted memory B cells in both HIV-1 and 2 infected groups (19, 55, 56). Evidence suggests that atypical memory B cells can efficiently present antigens to T cells (57). This is evident from the positive correlation found between atypical memory B cells and memory CD4^+^ T cells in our study. The expanded plasmablasts, atypical and memory cells are likely to be responsible for the increase in B cell turn over and slower disease progression associated with HIV-2 infection.

NK cells also influence the transition between innate and adaptive immune responses via the production of cytokines and chemokines (58). NK cell specificity for target cells and the ensuing effector functions are dependent on signalling from receptors that are expressed on the surface of NK cells (38, 59). Earlier studies have documented a dramatic increase in CD56^neg^ NK cells (60, 61) with low cytolytic, proliferative, and cytokine-producing capabilities in HIV-1 infection (28, 62, 63), that lyse HLA-I-deficient target cells and participate in antibody-dependent cytotoxicity (ADCC) (64, 65). Similarly, a subpopulation of CD56^dim^ NK cells called terminally matured NK cells express CD57, a marker that identifies antigen-experienced NK cells, was found to be elevated in elite controllers (66). In addition, NK cells expressing NKG2D, an activation receptor used for contacting target cells and subsequently resulting in the release of perforin and other cytotoxic molecules, were reported to be higher in these individuals (67). We found an increased frequency of CD56^dim^ NK cells as well as NKG2D expressing CD56^neg^ NK cells in HIV-2 infected individuals as compared to those with HIV-1 infection.

To summarize, HIV infection and the onset of AIDS is characterized by extensive interaction between the host immune system and the virus. The interaction results in profound quantitative and qualitative changes in both adaptive and innate immune cells, including T and B lymphocytes and natural killer cells in infected individuals. Both host genetic and viral factors are thought to contribute to non-progression of HIV disease (68), but the features of antiviral immunity that result in an effective immune response are only partially understood. The findings of the present study reveal that HIV-2 infection elicits a superior T cell response with high levels of critical T cell subsets including Tfh cells and stem cell-like CD4^+^ T cells that support the development of follicular homing T cells and other immune cell subsets and contribute to the control of infection. The study also documents robust memory B cell and NK cell responses that contribute to better control of HIV-2 infection and disease. We believe that vaccination strategies designed to elicit durable cellular immunity should target the generation of these immune cell types in order to provide adequate control of HIV infection. Further studies should be undertaken to fully understand the contribution and mechanistic role of these consequential cell types in HIV control.

## Data Availability Statement

The original contributions presented in the study are included in the article/**Supplementary Material**. Further inquiries can be directed to the corresponding author.

## Ethics Statement

The study protocol was also reviewed and approved by the Institutional Ethics Committee of ICMR-NIRT (TRC IEC No: 2009009) and the Institutional Review Board of the Y. R. Gaitonde Centre for AIDS Research and Education (YRG IRB No: 279), Chennai, India. The patients/participants provided their written informed consent to participate in this study.

## Author Contributions

SMP, VV and LH designed the conceptual framework of the study. SP, VV and NH performed the experiments. SMP analyzed the data. KT contributed to statistical analyses. KM contributed to specimen collection. MM and LH contributed to the review and editing of the manuscript. SP and VV wrote the manuscript. All authors contributed to the article and approved the submitted version.

## Funding

The present study was supported by the Department of Health Research (Human Resource Development Young Scientist Fellowship) and The Indian Council of Medical Research, Govt. of India.

## Conflict of Interest

The authors declare that the research was conducted in the absence of any commercial or financial relationships that could be construed as a potential conflict of interest.
