# Supplementary material for: Role of Circulating T Follicular Helper Cells and Stem-Like Memory CD4+ T Cells in the Pathogenesis of HIV-2 Infection and Disease Progression
Source: Front Immunol. 2021 Apr 16;12:666388. doi: 10.3389/fimmu.2021.666388 (PMC8085399; doi:10.3389/fimmu.2021.666388)
Supplement: Supplementary file 1 [file DataSheet_1.pdf]

Neutralization titre of HIV-2 study samples				
**Sample ID**	**7312A**	**HIV-2 NIRT010**	**MuLV**	**GMT**
NIRT-01	7380	391	<10	1699
NIRT-02	8185	290	<10	1542
NIRT-03	2440	3439	<10	2896
NIRT-04	7320	2295	<10	4098
NIRT-05	ND	ND	ND	NA
NIRT-06	18833	5480	<10	10158
NIRT-07	180	20	<10	61
NIRT-08	4980	2854	<10	3770
NIRT-09	2880	1260	<10	1904
NIRT-10	540	594	<10	566
NIRT-11	12150	118	<10	1199
NIRT-12	7236	8	<10	7236
NIRT-13	420	6801	<10	1690
NIRT-14	280	2052	<10	757
NIRT-15	180	216	<10	197
NIRT-16	11543	31000	<10	18916
NIRT-17	2520	900	<10	1505
NIRT-18	9180	2100	<10	4390
NIRT-19	10935	1371	<10	3871
NIRT-20	360	60	<10	146
NIRT-21	36453	14580	<10	23053
NIRT-22	180	180	<10	180
NIRT-23	12124	12765	<10	12440
NIRT-24	23858	159	<10	1948
NIRT-25	14580	510	<10	2726
NIRT-26	20655	450	<10	3048
NIRT-27	2520	58	<10	384
NIRT-28	10692	465	<10	2231
NIRT-29	12180	478	<10	2414
NIRT-30	3650	94	<10	588
NIRT-31	17921	103	<10	1361
NIRT-32	24723	140	<10	1860
NIRT-33	120	14580	<10	1322
NIRT-34	153	135	<10	143
NIRT-35	748	91	<10	261
NIRT-36	121	58	<10	84
NIRT-37	250	161	<10	200
